# A gain-of-function *Bushy dwarf tiller 1* mutation in rice microRNA gene *miR156d* caused by insertion of the DNA transposon *nDart1*

**DOI:** 10.1038/srep14357

**Published:** 2015-09-25

**Authors:** Mika Hayashi-Tsugane, Masahiko Maekawa, Kazuo Tsugane

**Affiliations:** 1 National Institute for Basic Biology, Okazaki 444-8585, Japan; 2 Institute of Plant Science and Resources, Okayama University, Kurashiki 710-0046, Japan; 3 The Graduate University for Advanced Studies [SOKENDAI], Okazaki 444-8585, Japan

## Abstract

A non-autonomous DNA transposon in rice, *nDart1*, is actively transposed in the presence of an autonomous element, *aDart1*, under natural conditions. The *nDart1*-promoted gene tagging line was developed using the endogenous *nDart1/aDart1* system to generate various rice mutants effectively. While the dominant mutants were occasionally isolated from the tagging line, it was unclear what causes dominant mutations. A semidominant mutant, *Bushy dwarf tiller1* (*Bdt1*), which has the valuable agronomic traits of multiple tillering and dwarfism, was obtained from the tagging line. *Bdt1* mutant carried a newly inserted *nDart1* at 38-bp upstream of transcription initiation site of a non-protein-coding gene, *miR156d*. This insertion caused an upstream shift of the *miR156d* transcription initiation site and, consequently, increased the functional transcripts producing mature microRNAs. These results indicate that the total amount of miR156d is controlled not only by transcript quantity but also by transcript quality. Furthermore, transgenic lines introduced an *miR156d* fragment that flanked the *nDart1* sequence at the 5′ region, suggesting that insertion of *nDart1* in the gene promoter region enhances gene expression as a cis-element. This study demonstrates the ability of *nDart1* to produce gain-of-function mutants as well as further insights into the function of transposable elements in genome evolution.

Although transposable elements consisting of highly repetitive DNA sequences were once thought to be junk DNA, they play an important role in genome reorganization and evolution^1^. In the 1940s, Barbara McClintock discovered the mobile element in maize, and various genome projects have revealed that transposable elements occupy large fractions of eukaryotic genomes. For example, approximately 10%, 35%, and 84% of Arabidopsis, rice, and Maize genomes, respectively, are transposon-derived sequences^2^,^3^,^4^. Active transposons are considered to be harmful to the host genomes because transposition induces chromosomal breakage and rearrangements and genetic changes. Only a limited number of transposons that escape from genetic or epigenetic regulation by host genomes can move^5^. Active transposons are often observed in plants as mutable phenotypes^6^,^7^,^8^.

Rice is an excellent model for the study on transposons because the whole genome sequences have been decoded with high accuracy and its genome is relatively transposon abundant^3^,^8^. An endogenous non-autonomous DNA transposon *nDart1-0* was identified in a rice *virescent* mutant (*Oryza sativa japonica* L., cv. Taichung 65 background), *pale-yellow leaf-variegated* (*pyl-v*)^9^. The *nDart1* elements are actively transposed over most of the genome in specific lines that carry an active autonomous element, *aDart1–27*, that encodes a transposase gene^10^. The *nDart1* elements, a member of *hAT* superfamily, excise from inserted chromosome sites and transpose to another site. *nDart1-0* and its closely related non-autonomous elements, *nDart1–1* to *nDart1–12*, exhibit different transposition frequencies in spite of their high sequence similarity^11^. Previously, we revealed that the most active element, *nDart1-0*, has a low cytosine methylation status, and the active *aDart1–27* possesses the hypomethylated promoter region^12^. Active transposons have been widely used as a tagging tool to elucidate gene functions. We developed the *nDart1/aDart1* tagging line using the endogenous DNA transposon *nDart1* elements. The tagging system is a powerful tool because *nDart1* elements are actively transposed under natural growth conditions and tend to be integrated into genic regions, particularly into promoter-proximal genic regions, 0.5 kb in front of the putative initiation codons^13^. To effectively identify *nDart1* insertion sites of the genome, the AFLP-based transposon display (TD) procedure, nDart-TD, and the iPCR-based procedure, nDart1-0-iPCR, were also developed^14^,^15^.

MicroRNAs (miRNAs) are important regulators of development in various organisms, including yeasts, fungi, animals, and plants^16^,^17^,^18^. In plants, transcripts transcribed from miRNA genes are processed into primary miRNAs (pri-miRNAs) with a 5′ cap and a poly-A tail. The pri-miRNAs form a stem-loop structure and then produce pre-miRNA by cleavage. The pre-miRNAs are processed into single-stranded small non-coding RNA molecules (~24 nucleotides). By interacting with the targeted genes, miRNAs induce post-transcriptional gene silencing. miRNAs were discovered by experimental approaches or bioinformatics predictions. To date, 592 miRNAs in 333 families have been annotated in rice (miRBase; http://www.mirbase.org/, Release 16). miR156 is well known as a quantitative repressor of vegetative phase change. miR156 targets *SQUAMOSA promoter-binding-like* (*SPL*) genes, and regulates the phase transition from the juvenile-to-adult phase^19^,^20^. The miR156 family is conserved in plants, and *miR156*-overexpressing transgenic rice, maize, Arabidopsis, and other plants demonstrated characteristic morphological changes: an increased number of branches, defects of the reproductive organs, a decrease in the number of seeds, and a reduction of tuber yields^21^,^22^,^23^,^24^. Overexpression of *miR156* reduces the plastochron length by suppressing *SPL* genes that delay juvenile-to-adult phase transition.

Several reports have described loss-of-function mutants isolated from the *nDart1*-promoted tagging line. Herein, we report a gain-of-function mutant designated *Bushy dwarf tiller1* (*Bdt1*) caused by insertion of *nDart1–3* 38 bp upstream of a non-coding RNA gene, *miR156d*. The *nDart1* insertion increased the functional *miR156d* transcription level and conferred the multiple tillering and dwarf phenotype. The mechanism of the gain-of-function mutant is not well known so far. This study provides further insight into the function of active transposons and also clues regarding the mechanism of gain-of-function mutations and genome evolutions.

## Results

### Phenotype of semidominant *Bdt1* mutant

**Figure 1:**
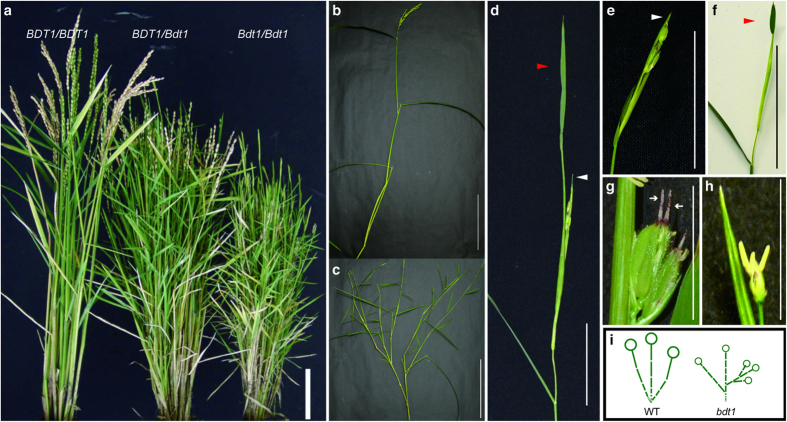
Semidominant phenotypes of *Bdt1* plants. (**a**) Three-month old wild-type (*BDT1/BDT1*), *Bdt1* heterozygous (*BDT1/Bdt1*), and *Bdt1* homozygous (*Bdt1/Bdt1*) plants. Bar = 10 cm. Primary culms of the WT (**b)** and *Bdt1/Bdt1* plants (**c**). Bar = 10 cm. (**d**–**h**) Abnormal panicles of *Bdt1/Bdt1* plants. Bar = 5 cm in D–F, bar = 1 cm in G and H. White and red arrowheads indicate overgrown bracts and leaf-like structures, respectively. (**i**) Morphological phenotypes of WT and *Bdt1* plants. Each broken line and each circle represents an internode and a panicle, respectively.

A semidwarf mutant with an increasing number of tillers, designated as *Bdt1*, was isolated from the *nDart1*-promoted tagging line. A founder mutation in 07nD45-4 observed as normal or short panicles in individual tillers. The next generation plants that were derived from 13 panicles in independent tillers in 07nD45-4 were grown. Further genetic analysis showed that *Bdt1* is a semidominant mutation for bushy and dwarf phenotypes in a single locus (Table 1). The homozygote mutant exhibited a more severe phenotype than that of the heterozygous mutant (Fig. 1a). The *Bdt1* plants showed a bushy phenotype by developing secondary branches from irregular positions of culms. Although the internode length gradually increases from the basal to the upper part of the culm in rice (Fig. 1b), *Bdt1* plants had nearly equal lengths of internodes (Fig. 1c). As compared with the wild-type plant, *Bdt1* plants developed smaller leaves and more compact panicles. The homozygous *Bdt1* plant bore fewer spikelets, and some panicles were morphologically aberrant and sterile. Overgrown bracts and leafy organs were observed above the panicle nodes (Fig. 1d–f). Some of the panicles appeared not to be exerted (Fig. 1f). Generally, a spikelet develops an awn from the lemma, but a pair of awns developed in the abnormal *Bdt1* spikelet (Fig. 1g). A spikelet without a lemma and palea was also observed (Fig. 1h). The morphological differences between wild-type and *Bdt1* plants are schematically represented in Fig. 1i.

### Identification of the *Bdt1* allele

**Figure 2:**
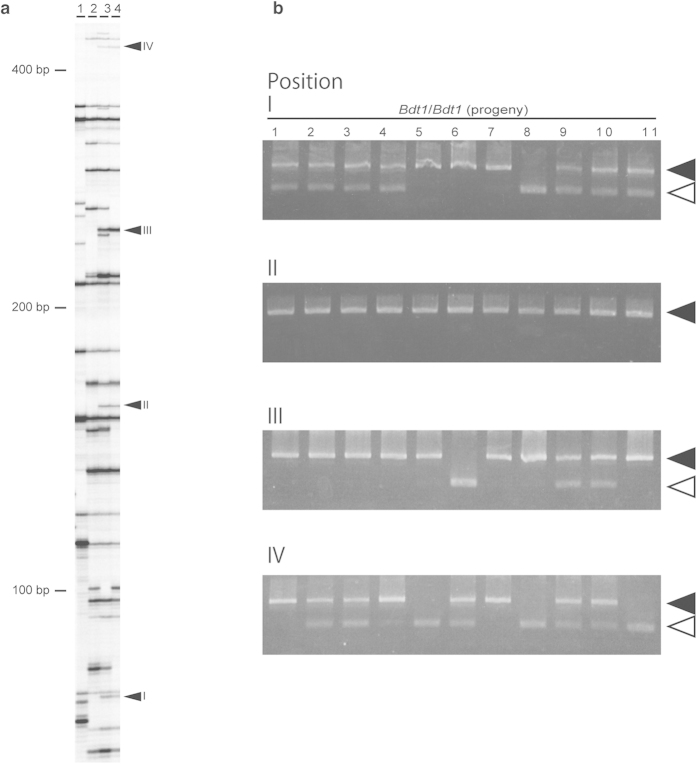
Identification of the *Bdt1* allele. (**a**) TD analysis with *TaqI*-digested DNA are lane 1, Nipponbare; lane 2, *BDT1/BDT1*; lane 3, *BDT1/Bdt1*; lane 4, *Bdt1/Bdt1*. 1 The arrowheads represent polymorphic bands (positions 1–4) among WT and *Bdt1* plants. (**b**) Genotyping of progeny plants obtained from *Bdt1/Bdt1* by PCR analysis. The filled and open arrowheads indicate PCR-amplified bands using primer sets that detect *nDart1–3* insertion at positions I–IV. Insertion of *nDart1* was detected in all of the progeny only at position II among four candidates.

**Figure 3:**
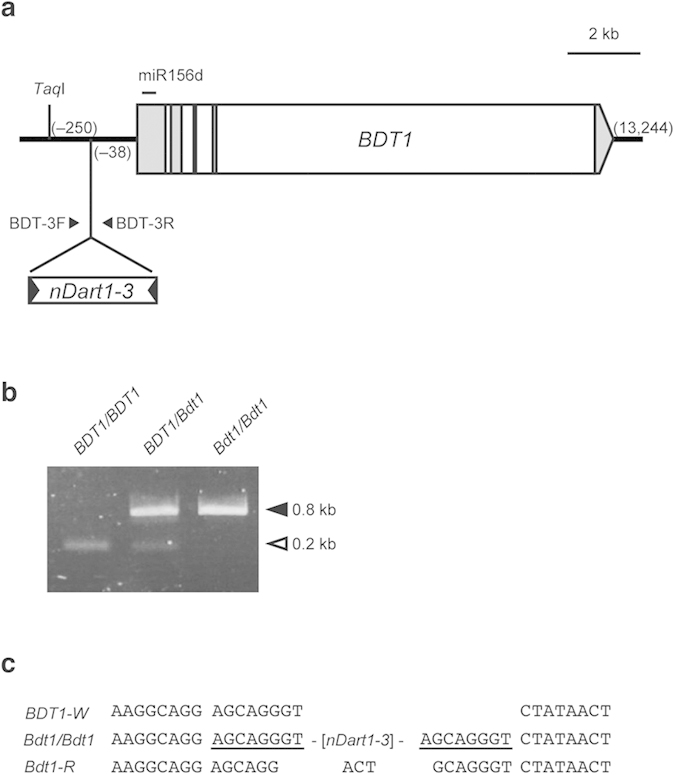
Characterization of the *Bdt1* allele. (**a**) Structure of the *BDT1* gene carrying the *nDart1–3* insertion. Shaded and white boxes represent *BDT1* exons and introns, respectively. The horizontal line above the box represents the position where mature miR156d is transcribed. The numbers in parentheses represent the nucleotide positions from the transcription initiation sites of full-length cDNA AK073452. The small horizontal arrowheads indicate positions of the primers used for PCR amplification to characterize the insertions of *nDart1–3* (Fig. 2b). (**b**) Genotyping of WT and *Bdt1* plants by PCR analysis. The filled and open arrowheads indicate PCR-amplified bands with and without *nDart1* insertion, respectively. (**c**) Sequence of the footprints generated by *nDart1–3* excisions. The WT, homozygous *Bdt1*, and germinal revertant alleles are indicated by *BDT1-W*, *Bdt1/Bdt1*, and *Bdt1-R*, respectively. Target site duplication generated by *nDart1–3* insertion at the *Bdt1* locus is underlined.

Because we predicted that a new insertion of the *nDart1* element caused the *Bdt1* mutation, nDart-TD was performed to identify unique insertion sites of *nDart1* elements in *Bdt1* plants. Using nDart-TD, 4 newly inserted *nDart1* elements were detected as common bands between *Bdt1/Bdt1* and *BDT1/Bdt1* mutants (Fig. 2a). To eliminate non-*Bdt1*-related insertions, the genotype of the 4 genes was identified among the *Bdt1* progeny. The results showed that the *Bdt1* phenotype associated with the insertion was band II (Fig. 2b). This new insertion was *nDart1–3*, one of the active *nDart1* elements. We identified the new insertion 38 bp upstream of the transcription initiation site (TIS) of the *Os02g0180800* gene (AK073452, full-length cDNA in Nipponbare). This gene consists of five exons, and the first exon contains a 22-nt sequence corresponding to mature miR156d (Fig. 3a). Subsequent PCR analysis with primers flanking the *nDart1–3* revealed that the *nDart1–3* insertion in the *Bdt1/Bdt1* plants was in the homozygous condition, and the amplified fragments from the *BDT1/Bdt1* plant were identical in size to those from wild-type and *Bdt1/Bdt1* plants (Fig. 3b). The mutant phenotype cosegregated with the *nDart1–3* insertion. These results suggest that *nDart1–3* insertion upstream of *miR156d* conferred the *Bdt1* mutant phenotype.

Excision events of DNA transposons often leave sequence alternation called footprint at the flanking sites. Both perfect excision of the transposon sequence and a footprint that does not impair gene function(s) can generate revertants (i.e., nucleotide additions or deletions in multiples of three nucleotides generate wild-type revertants). *nDart1*-promoted mutants often produce germinal revertants in their progeny. To assess whether the *Bdt1* mutation was indeed caused by the insertion of *nDart1–3*, we isolated a germinal revertant with a wild-type phenotype and examined the excision of *nDart1–3* from the *Bdt1* allele. The germinal revertant with the *Bdt1-R* allele was found to carry an 8-bp insertion and nucleotide substitutions as the footprint (Fig. 3c). Based on these results, we can conclude that the *BDT1* gene corresponds to a non-coding RNA gene, *Os02g0180800*.

### Transcript analysis of *Bdt1*

**Figure 4:**
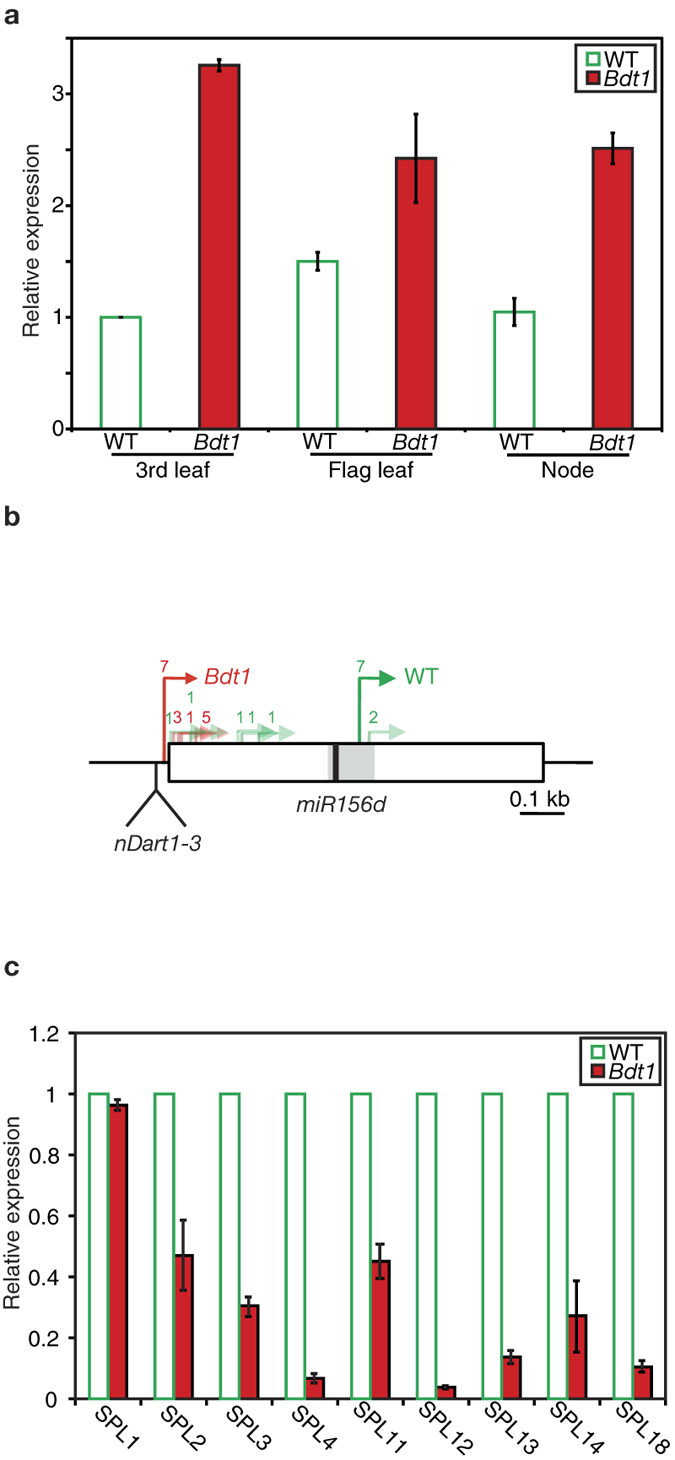
Transcription analysis of functional *miR156d*. (**a**) Tissue-specific expression patterns of the *miR156d* gene in WT and *Bdt1* plants. (**b**) Major transcription initiation sites of *miR156d* in WT and *Bdt1* plants. Red and green arrows indicate transcription initiation sites of *miR156d* in WT and *Bdt1* plants, respectively. Numbers above the arrows represent the numbers of clones that correspond to the transcription initiation site. The left end of the white box indicates the reported 5′ terminal of the full-length cDNA (AK073452) in Nipponbare (http://rapdb.dna.affrc.go.jp/). The gray and black boxes show the corresponding positions of the *pre-miR156d* and *miR156d* sequences. (**c**) Relative expression of *SPL1* gene and miR156-targeted *SPL* genes in 3rd leaves of WT and *Bdt1 plants*. Error bars indicate SD.

*nDart1–3* insertion upstream of *miR156d* caused a semidominant phenotype. To investigate the effects of *nDart1–3* insertion on the expression of the *miR156d* gene, real-time quantitative PCR (qPCR) analysis was performed. The expression levels of *microRNA* genes vary according to the developmental stage and the tissue type. We evaluated the expression levels in 3rd leaves, flag leaves, and lowermost nodes of *Bdt1/Bdt1* plants using a primer set to specifically amplify the 5′ region of *BDT1* where miR156d corresponding sequence. The results showed that the *miR156d* expression level in 3rd leaves of *Bdt1/Bdt1* seedlings was more than 3-fold higher than that of wild-type seedlings (Fig. 4a). *miR156d* was also highly expressed in the flag leaves and nodes of mutants (Fig. 4a). *nDart1–3* insertion increased the *miR156d* transcripts containing pre-miR156d through juvenile and adult stages in *Bdt1* plants.

In order to clarify the regulation mechanism of *miR156d* expression in *Bdt1* mutants, transcription initiation sites (TIS) of the *miR156d* gene in wild-type and *Bdt1/Bdt1* plants were identified by 5′-RACE analysis. In leaf blades of wild-type seedlings, some of the transcription started from a few different sites upstream of the pre-miR156d region, but the major TIS was downstream of the mature miR156d sequence. In contrast, the TISs in leaf blades of *Bdt1/Bdt1* seedlings were only detected upstream of the pre-miR156d region. All *Bdt1* transcripts contained the pre-miR156d region. The detected TISs are summarized in Fig. 4b. These results suggest that *nDart1–3* insertion altered the TIS of *miR156d*, and the upstream shift in the transcripts increased functional *miR156d* expression.

### Expression of miR156d-targeted *SPL* genes

In rice, 11 of 19 *SPL* genes contain miR156 target sites^23^. To confirm an increase of mature miR156d levels in *Bdt1*, the expression levels of miR156d-targeted *SPL* genes were examined in the 3rd leaves of wild-type and *Bdt1* seedlings. qPCR analysis showed that a non-miR156d-targeted gene, *SPL1*, was expressed at the same level in wild-type and *Bdt1* seedlings (Fig. 4c). In contrast, expression levels of miR156d-targeted *SPL*s (*SPL2*, *SPL3*, *SPL4*, *SPL11*, *SPL12*, *SPL13*, *SPL14*, and *SPL18*) were significantly decreased in *Bdt1* seedlings (Fig. 4c). The expression levels of *SPL4* and *SPL12* were reduced more than 90%. These results suggest that *BDT1* transcripts induced by *nDart1–3* insertion generate functional microRNA and effectively suppress expression of *SPL* genes.

### Effects of *nDart1* insertion as a cis-element

**Figure 5:**
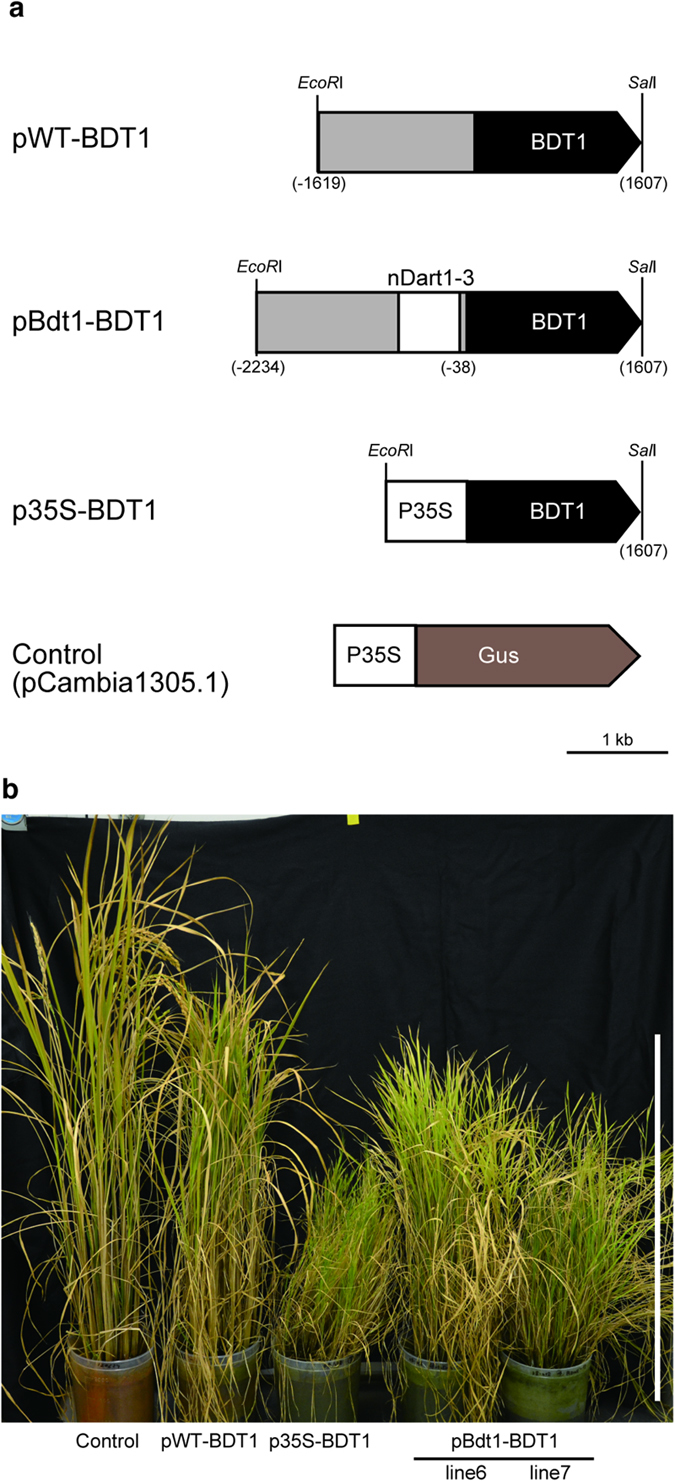
Effects of *nDart1–3* insertion on *miR156d* expression. (**a**) Constructs of plasmid vectors used to generate transgenic lines. (**b**) Three-month old transgenic T0 lines. Scale bar = 50 cm.

Expression analysis of *Bdt1* plants revealed that *nDart1–3* insertion activated its downstream gene expression. To evaluate the effects of *nDart1–3* on its downstream gene, we constructed three types of vectors, generated rice transgenic lines, and compared the phenotypes. Vectors pWT-BDT1 and pBdt1-BDT1 carried 1.6-kb *BDT1* cDNA sequences (AK073452) that flanked the upstream region of wild-type and *Bdt1* plants, respectively (Fig. 5a). Both constructs were postulated to contain the *BDT1* promoter region. In pBdt1-BDT1, the 607-bp *nDart1–3* sequence presents within the promoter region (Fig. 5a). Transgenic lines with CaMV35S promoter-driven *BDT1* were generated to evaluate *Bdt1* expression levels (Fig. 5a). By *Agrobacterium*-mediated transformation with 10 pCambia1305.1 (Control), 8 pWT-BDT1 lines, 12 pBdt1-BDT1 lines, and 10 p35S-BDT1 lines were obtained (Supplemental Table S1). Typical examples of T0 plants are shown in Fig. 5b. Transgenic plants transformed with pWT-BDT1 were moderately short. pBdt1-BDT1 and p35S-BDT1 plants exhibited morphological changes observed in plants that overexpressed *miR156*. The pBdt1-BDT1 lines had aberrant panicles and significantly reduced fertility. p35S-BDT1 transgenic lines showed more significant changes in phenotype. p35S-BDT1 plants generated no T1 generation due to the lack of inflorescences and a sterile spikelet on a branch. These observations indicate that the *nDart1–3* sequence indeed increased the amount of mature miR156d in *Bdt1,* without disruption of *miR156d* gene. The miR156 function is quantitative. Based on the phenotype, the effect of *nDart1–3* on *miR156d* expression was modest as compared to that of the CaMV35S promoter.

## Discussion

A semidominant mutant *Bdt1* that displayed a semidwarf phenotype, enhanced tillering, and compact (and aberrant) panicles was isolated from the *nDart1*-promoted tagging line (Fig. 1). nDart-TD analysis revealed that the mutant allele *Bdt1* was caused by the insertion of *nDart1–3* into the promoter region of the microRNA gene, *miR156d* (Fig. 3a). Afterward, the mutant allele was confirmed by the fact that the germinal revertant exhibited the wild-type phenotype. The expression level of the *miR156d* transcripts in leaf blades of *Bdt1* seedlings was approximately threefold higher than that of the wild type (Fig. 4a). 5′-RACE analysis showed that *miR156d* transcripts without mature miR156d sequences were not a small amount in wild-type leaves. On the other hand, all *miR156d* transcripts contained a pre-miR156d region, and the major TIS was relatively upstream in *Bdt1* leaves (Fig. 4b). These results suggest that *miR156d* transcripts were originally a mixture that included and excluded the pre-miR156d region. The upstream shift of the TIS by *nDart1–3* insertion increased the functional transcripts that produced mature miR156d. The quantity of functional *miR156d* transcripts is controlled not only by the transcription activity but also by the TIS of *miR156d*. The *miR156d* would have a complex regulation mechanism for the generation of mature miR156d. The major TIS may vary based on the plant organ and the developmental stage in plants.

In rice, the miR156 family consists of 12 members (*a–l*), and *miR156* genes (*a–j*) produce the identical 20-nt mature miR156^25^,^26^. These microRNA genes show different spatial and temporal patterns of expression and different interaction patterns with *SPLs* in spite of the total match of miRNA sequences^26^. To confirm the increase of mature miR156d in *Bdt1*, the expression levels of miR156d-targeted *SPLs* were examined. RT-qPCR analysis showed that the expression of the targeted *SPLs* was obviously decreased. These results suggest that the increased *miR156d* transcripts in *Bdt1* seedlings were efficiently processed into mature microRNA and indeed suppressed the targeted genes. The reduced ratio in a series of *SPLs* was not equal, but these patterns were similar to the results in *miR156d*-overexpressing plants^26^. The suppression level of *miR156d* varies according to the organ and the developmental stage. *miR156d* would not primarily contribute to the regulation of *SPL2* and *SPL11*.

Subsequently, to evaluate the effects of *nDart1–3* insertion on downstream gene expression, three types of transgenic plants were constructed. As part of a complementation assay, a *BDT1* fragment that flanked the wild-type promoter region and a *BDT1* fragment that flanked the *Bdt1* upstream region were introduced into Nipponbare (Fig. 5a). The transgenic lines indicate that the *nDart1* sequence in the promoter upregulated *miR156d* expression. The pBdt1-BDT1 lines had aberrant panicles and significantly reduced fertility. The CaMV35S-driven transgenic lines exhibited a more severe phenotype that resulted in complete sterility. The effect of *nDart1* insertion was lower than that of a constitutive promoter, CaMV35S. The modest semidwarf phenotype in pWT-BDT1 lines can be interpreted as the co-expression of endogenous and exogenous *BDT1* gene(s). Our previous study, using *GUS* reporter genes, showed that *nDart1* itself does not have promoter activity^10^. Therefore, *nDart1* elements require an endogenous promoter to upregulate flanking gene expressions. The increase of miR156d in *Bdt1* mutation would mainly result from TIS alternation by recognition of RNA polymerase II. Generation patterns of miR156d in endogenous promoter-dependent *Bdt1* plants must be different from those of constitutive promoter-driven *miR156d*-overexpressing plants in developmental stages and tissue types.

Several reports described gain-of-function mutants caused by transposons. The *teosinte branched1* (*tb1*) gene in maize was identified as the major contributor to domestication of modern corn from the grass-like ancestor teosinte (*Zea mays* ssp. *parviglumis*)^27^. Recent studies have revealed that insertions of *LTR-retrotransposon, Hopscotch* 58–69 kb upstream of *tb1* are associated with a quantitative increase in the expression of the maize *tb1* allele^28^,^29^. Further genetic analysis demonstrated that the expression level in maize with transposon insertions upstream of the *tb1* regulatory region was higher than that of teosinte. *Cg1* is a maize *miR156*-overexpressing mutant caused by the insertion of a retrotransposon, *STONER*, 42 bp upstream of the TIS of tandem *miR156b/c* genes^21^. *Bdt1* and *Cg1* appeared to be the results of similar events in the genes and transposon insertion sites. However, *nDart1–3* is a non-autonomous element that does not encode transposase, and the TIS of *miR156d* was downstream of *nDart1–3*. On the other hand, *STONER* is a long autonomous element, and *Cg1* transcription is initiated within the *STONER* element. The activation mechanism of the downstream gene by *nDart1* insertion must be different from that of *Cg1*. In the case of a dominant rice mutant *apo1-D*, *nDart1-0* was inserted 3.5 kb upstream of the *APO1* region and caused an increase of *APO1* expression^30^. They suggested that the *nDart1-0* insertion conferred the loss of negative regulation of *APO1* expression. Because the *nDart1* insertion sites were not adjacent to the *APO1* TIS, the *nDart1* must disrupt the silencer region of *APO1*.

The *nDart1*-promoted tagging line is highly expected to produce both loss-of-function and gain-of-function mutants to characterize various unknown genes because uncharacterized gene functions by loss-of-function mutation may be clarified by studying gain-of-function mutants. For example, *pyl* and *snow-white leaf1* are recessive loss-of-function mutants caused by *nDart1* insertion into the 5′UTR. These insertions caused a downshift of the TIS, and their transcripts lacked the proper initiation codons^9^,^15^. In *Bdt1*, *nDart1* caused an upstream shift of the TIS and increased gene expression, including the pre-miR156d region. However, the mechanism of gain-of-function mutation is not well understood. Further investigation of *nDart1* insertion sites and their impact on gene expression are necessary to reveal the alternation mechanism of TIS. It is well known that transposons are important sources of evolution. Some transposons can enhance neighboring gene expressions^30^,^31^,^32^. On the other hand, *nDart1* elements did not have such promoter/enhancer activity^10^, but this study demonstrated that transposons without such activity contribute to genome evolution and genetic variation not only by gene disruption but also by activating gene expressions. We believe that this valuable feature of *nDart1* will promote fine-tuning the useful properties for breeding.

## Materials and Methods

### Plant materials and growth conditions

Plants were grown in a growth chamber (12 h of light and 12 h of darkness) at 30 °C or in pots under natural conditions as previously described^9^.

### Identification of the *Bdt1* allele by nDart-TD

The nDart-TD was carried out according to the procedure previously reported with some modifications^14^. The genomic DNA was cleaved with the *Taq*I restriction enzyme and ligated to the adapter in a 25-μl reaction mixture. The initial amplification was performed in a 20-μl reaction mixture that contained adapter-ligated DNA and a set of adapter and Dart5′-1-1st primers. Subsequently, a set of primers composed of the adapter primer and the Dart5′-1,2,3-2nd primer labeled with 6-carboxyfluorescein (4.8 pmol each) was subjected to nested amplification. The 0.5 μl of amplified fragments mixed with a loading cocktail containing 9 μl of Hi-Di Formamide was heated at 95 °C for 3 min and then chilled on ice. The samples injected into an ABI 3130xl genetic analyzer (Applied Biosystems, Foster City, CA, USA) were analyzed by using GeneMarker (SoftGenetics, LLC, State College, PA, USA) genotyping software to detect TD patterns. To read the nucleotide sequences of the candidate *nDart1* integration sites where wild-type and *Bdt1* plants show polymorphism, the rest of the amplification products were concentrated by ethanol precipitation and electrophoresed on a 5% denaturing polyacrylamide gel. The bands were visualized using FMBIO II Multi-View (Hitachi Software Engineering, Tokyo, Japan). Unique bands in the *Bdt1* mutants were extracted from the gel and were amplified with a set of adapter and Dart5′-1,2,3-2nd primers. The reaction products were subjected to sequencing analysis. The primer and adapter sequences used for TD analysis are listed in Supplemental Table S2.

### General nucleic acid procedures

General nucleic acid procedures, including the preparation of genomic DNA and RNA, PCR and RT-PCR amplifications, and 5′-RACE analysis, were performed as previously described^9^,^15^.

### RT-qPCR

Primers used to examine mRNA expressions are listed in Supplemental Table S2. Real-time RT-qPCR for expressions of *SPL* genes was performed as described in Xie *et al.* 2012. The expression levels of *miR156d* and *SPL* genes were normalized to the reference genes U6 (RNA) and AK071592, respectively, and their relative expression levels were calculated using the 2^−ΔΔCT^ method^31^. Each set of primers was tested with cDNA from wild-type and *Bdt1/Bdt1* plants with three technical^32^,^33^ replicates per sample using the THUNDERBIRD SYBER qPCR Mix (Toyobo, Osaka, Japan).

### Constructs and transformation

For p35S-BDT1, the AK073452 fragment carrying *Pst*I*-Sal*I sites was obtained by amplification of full-length cDNA of AK073452 (the Rice Genome Resource Center RGRC, http://www.rgrc.dna.affrc.go.jp/index.html), using primers AK073452-PstI and AK073452-SalI, and the fragment was fused to the CaMV35S-promoter sequence carrying *EcoR*I-*Pst*I sites amplified with primers 35S-EcoRI and 35S-XhoI. For pWT-BDT1 and pBdt1-BDT1, the AK073452 fragment carrying *BamH*I-*Sal*I sites was fused to 1.6- and 2.2-kb fragments carrying *EcoR*I*-BamH*I sites amplified from the AK073452 upstream regions of wild-type and *Bdt1* plants with primers BDT1-F (−) 1616-EcoRI and BDT1-R177, respectively. These three fragments were introduced into multiple cloning sites of the pCAMBIA1305.1 vector. As a negative control, the pCAMBIA1305.1 vector was used.

*Agrobacterium*-mediated rice transformation was performed using *Agrobacterium tumefaciens* strain EHA105, as described previously^10^.

## Additional Information

**How to cite this article**: Hayashi-Tsugane, M. *et al.* A gain-of-function *Bushy dwarf tiller 1* mutation in rice microRNA gene *miR156d* caused by insertion of the DNA transposon *nDart1*. *Sci. Rep.* **5**, 14357; doi: 10.1038/srep14357 (2015).

## Supplementary Material

Supplementary Information

## Figures and Tables

**Table 1:** Segregation of dwarf and semi-dwarf phenotype in *Bdt1* mutant progeny.

Panicle-row lines	Panicle type in a founder	Segregation in next generation		
		Normal	Intermediate	Mutant
3	Short	2	5	3
4	Short	1	5	2
5	Short	1	2	2
6	Short	1	3	4
7	Short	4	3	2
8	Short	2	2	3
9	Short	2	5	3
10	Short	2	2	1
11	Short	2	4	2
12	Short	1	2	1
	Total*	18	33	23
1	Normal	10	0	0
2	Normal	10	0	0
13	Normal	10	0	0
	Total	30	0	0

^*^Chi-square value = 1.54, df = 2, 0.25 < P < 0.50
