# Supplementary material for: A gain-of-function Bushy dwarf tiller 1 mutation in rice microRNA gene miR156d caused by insertion of the DNA transposon nDart1
Source: Sci Rep. 2015 Sep 25;5:14357. doi: 10.1038/srep14357 (PMC4585910; doi:10.1038/srep14357)
Supplement: Supplementary Information [file srep14357-s1.pdf]

Supplementary Data

A gain-of-function *Bushy dwarf tiller 1* mutation in rice microRNA gene *miR156d* caused by insertion of the DNA transposon *nDart1*

Mika Hayashi-Tsugane[1,4], Masahiko Maekawa[2], and Kazuo Tsugane[1,3,*]

[1]National Institute for Basic Biology, Okazaki 444-8585, Japan

[2]Institute of Plant Science and Resources, Okayama University, Kurashiki 710-0046, Japan

[3]The Graduate University for Advanced Studies [SOKENDAI] Okazaki 444-8585, Japan

Present address

[4]Graduate School of Bioagricultural Sciences, Nagoya University, Chikusa, Nagoya, Japan

* Corresponding author; fax: (+81) 564 557521, E-mail: tsugane@nibb.ac.jp

Supplementary Table S1. Phenotypes of T0 generation in complementaion test

		Control	pWT	p35S	pBdt
Plant nmber		10	8	10	12
Height	~75~	10		1	
(cM)	~65		6	1	6
	~55		1	1	
	~45		1		4
	~35		1*		2*
	~25			7*	

*Sterile

Supplementary Table S2. List of primer sequences used in this study

Use	Name	Sequence (5' to 3')
OsmiR156d precursor specific primer	BDT-3F	TCTCGATTTCGATGACTCGG
	BDT-3R	GCGAGGACGGGATAGATTTTA
5'-RACE	AK073452-R493	GTAATTTAGTGATTTTGTAAGCC
Vectors construction	AK073452-PstI	GGGCTGCAGGAGGTACGACCATCACCC
	AK073452-SalI	CCCGTCGACTGGCCGGCTCCGGACTGTTT
	35S-EcoRI	CCCGAATTCATGGAGTCAAAGATTCA
	35S-XhoI	GGGCTGCAGTCCCCCGTGTTCTCTCC
	BDT1-F(-)1616-EcoRI	CCCGAATTCATACCCTTGGTAGCAC
	BDT1-R177	AAGAGAGGTTTTCTTCTTGGATCCG
